# Structural elucidation of a hy­droxy–cineole product obtained from cytochrome P450 monooxygenase CYP101J2 catalysed transformation of 1,8-cineole

**DOI:** 10.1107/S2056989017010015

**Published:** 2017-07-21

**Authors:** Gavin E. Collis, Birgit Unterweger, Geoff J. Dumsday, Craig M. Forsyth

**Affiliations:** a Advanced Fibres and Chemical Industries, CSIRO Manufacturing, Melbourne, Victoria 3169, Australia; b Infection and Immunity Program, Monash Biomedicine Discovery Institute and Department of Microbiology, Monash University, Clayton 3800, Australia; c School of Chemistry, Monash University, Clayton 3800, Australia

**Keywords:** crystal structure

## Abstract

1,8-Cineole is an abundant natural product that has the potential to be transformed into other building blocks that could be suitable alternatives to petroleum-based chemicals. Mono­hydroxy­lation of 1,8-cineole can potentially occur at eight different carbon sites around the bicyclic ring system. Using cytochrome P450 monooxygenase CYP101J2 from *Sphingobium yanoikuyae* B2, the hy­droxy­lation can be regioselectively directed at the C atom adjacent to the methyl-substituted quaternary bridgehead atom of 1,8-cineole. The unambiguous location of the hydroxyl functionality and the stereochemistry at this position was determined by X-ray crystal analysis. The mono­hydroxy­lated compound derived from this microorganism was determined to be (1*S*)-2a-hy­droxy-1,8-cineole (trivial name) or (1*S*,4*R*,6*S*)-1,3,3-trimethyl-2-oxabi­cyclo­[2.2.2]octan-6-ol (V) (systematic), C_10_H_18_O_2_. In the solid state this compound exhibits an inter­esting O—H⋯O hydrogen-bonding motif.

## Abstract 2

X-ray structure analysis of hy­droxy–cineole, derived from the biotransformation of cineole, was undertaken to unambiguously determine the location and stereochemistry of the hydroxyl functionality. In the solid state, weak intra­molecular O—H⋯O hydrogen bonding is present, causing the mol­ecules to arrange in spiral chains.

## Chemical context   

**Figure 1:**
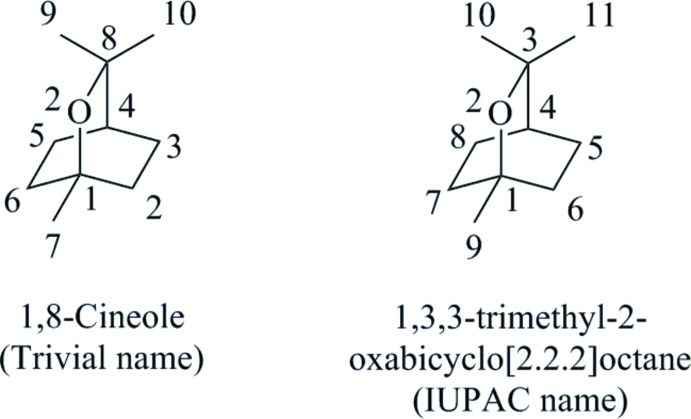
Trivial and systematic naming and atom numbering used for compound (I).

The terpenoid compound commonly known as 1,8-cineole, or less easily identified using systematic nomenclature as 1,3,3-trimethyl-2-oxabi­cyclo­[2.2.2]octane (I) (Fig. 1 ▸), is a key component of the leaf oil from eucalypts and is also found in a variety of plant types, such as sage, thyme and fruit extracts, albeit in lower qu­anti­ties (Fig. 1 ▸). Its natural abundance makes it a suitable bio-derived feedstock from which other useful chemical building blocks could be accessed and used as an alternative to petrochemical based-materials. Although continued research into the chemical and biochemical transformation of 1,8-cineole (I) is being directed towards accessing high quality and commercial qu­anti­ties of these derivatives, the naming of these products by using non-systematic nomenclature, coupled with the chiral nature of these products has created inconsistencies and made it challenging to compare data of these derivatives in the literature. To address this Azerad (2014 ▸) recently published an extremely useful review article capturing all the oxidation products of 1,8-cineole (I) by providing trivial and systematic names along with characterization data (i.e. melting point, optical rotation and proton and carbon NMR spectroscopic information).

**Figure 2:**
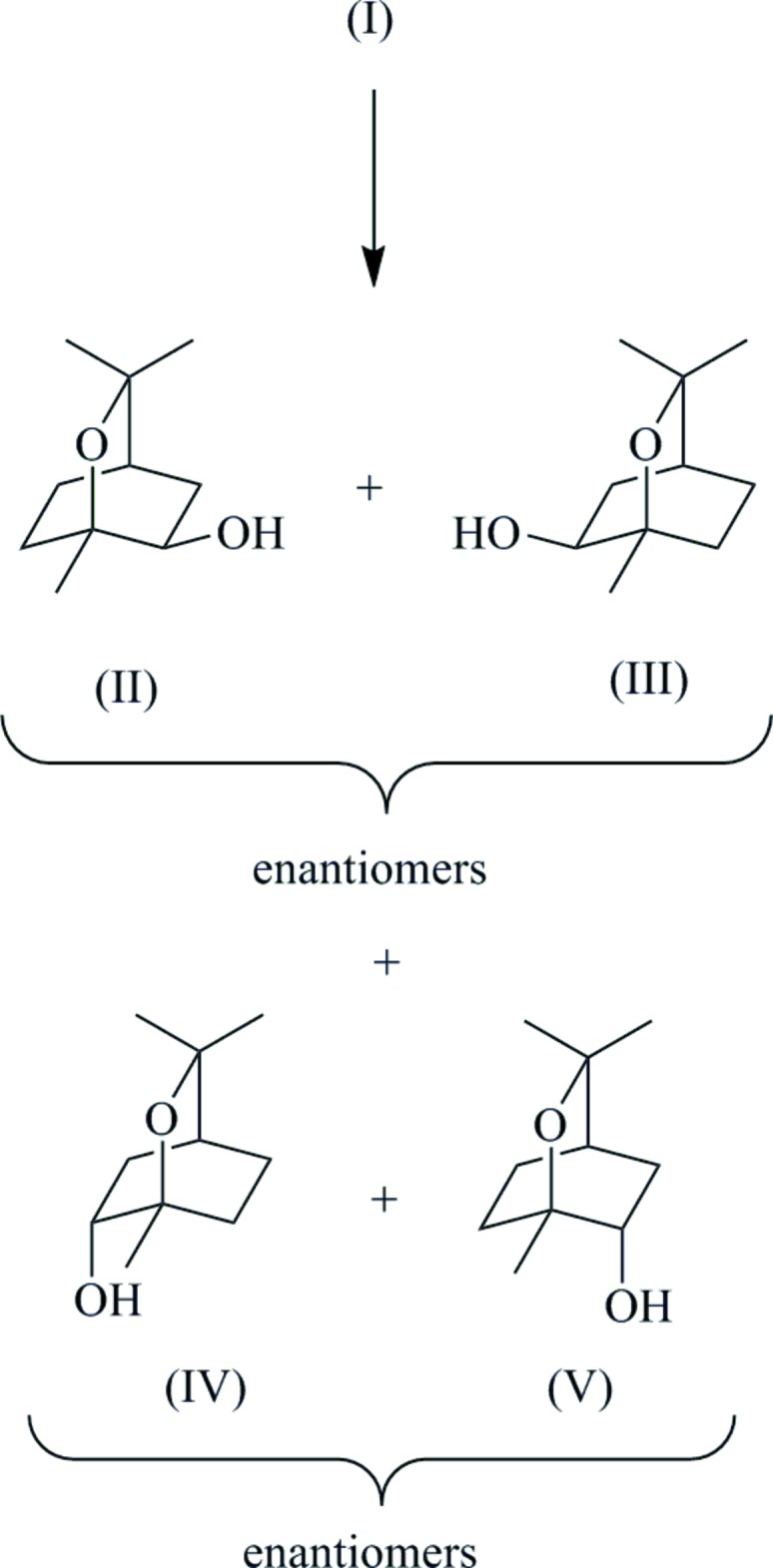
Biotransformation of 1,8-cineole (I) by *S. yanoikuyae* B2 to produce four possible isomeric mono-hy­droxy­lated products (Unterweger *et al.*, 2016 ▸).

In continuing our research activities on the biocatalytic mono-hy­droxy­lation of 1,8-cineole (I) at the C atom adjacent to the quaternary C1 bridgehead atom (*i.e*. labelled 6 or 7 following IUPAC rules) four possible stereoisomers [Fig. 2 ▸, compounds (II), (III), (IV) and (V)] could be formed. However, there is no current crystallographic information of these pure materials to support these assignments. Knowing the inconsistencies with the nomenclature of these compounds and to gain a better understanding of how to control the regio- and stereo-chemistry at the different sites around the 1,8-cineole bicyclic ring system, we sought confirmation of the absolute configuration by undertaking X-ray crystallographic studies.

## Structural commentary   

**Figure 3:**
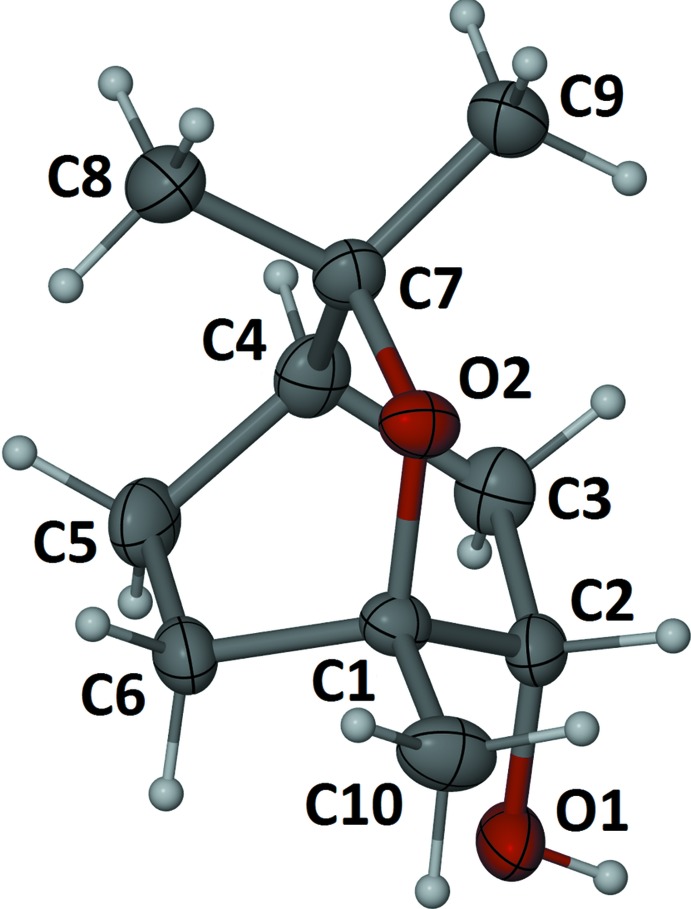
Mol­ecular structure of (1*S*,4*R*,6*S*)-1,3,3-trimethyl-2-oxabi­cyclo­[2.2.2]octan-6-ol (V) with non-H atoms represented by 50% displacement ellipsoids and H atoms as spheres of arbitrary size.

Suitable crystals for X-ray diffraction were prepared by the slow diffusion of petroleum ether into a solution of the compound dissolved in ethyl acetate. The X-ray crystal structure of the purified mono-hy­droxy­lated 1,8-cineole (V) Fig. 3 ▸) was solved in the *P*2_1_ space group and revealed the location of the hydroxyl group to be in the 6 position (IUPAC) (Fig. 1 ▸). The absolute configuration was determined by the method of Parsons *et al.* (2013 ▸) and confirmed the proposed stereochemistry (*i.e.* structure (V) see above, Fig. 2 ▸).
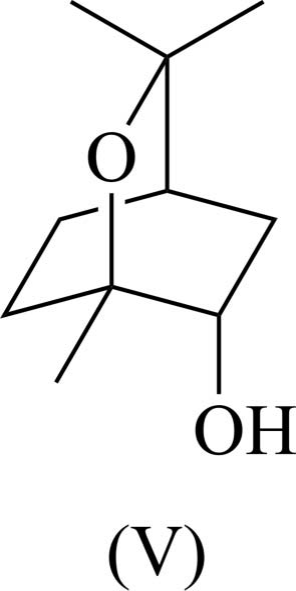


The presence of the axial hydroxyl substituent in (V) breaks the crystallographic symmetry of the parent 1,8-cineole (I), with *P*2_1_/*m* space group (Bond & Davies, 2001 ▸), resulting in a slight twisting of the mol­ecular framework as shown by the torsion angle C1—O2—C7—C4 of −12.8 (2)° and is presumably steric in origin. For the related 1,8-cineole-5,6-diol, three of the four possible diastereoisomers have been structurally characterized and only the one with the 6α hydroxyl group showed a similar distortion (Farlow *et al.*, 2013 ▸).

## Supra­molecular features   

**Figure 4:**
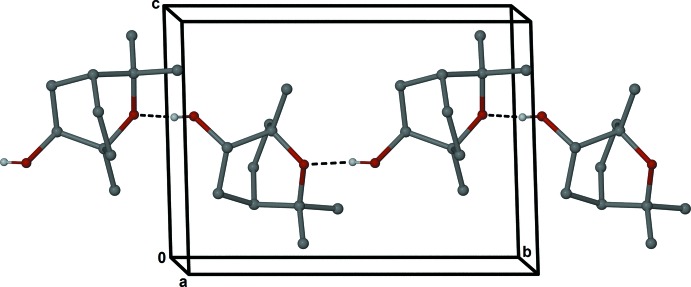
Ball-and-stick representation of a hydrogen-bonded chain of mol­ecules of (V). Only selected H atoms are shown and O—H⋯O contacts are indicated as dashed bonds.

Individual mol­ecules of (V) are connected by O–H⋯O hydrogen bonds between the hydroxyl and ether moieties (Table 1 ▸) and form spiral chains parallel to the *b* axis (Fig. 4 ▸).

## Database survey   

A search of the Cambridge Structural Database (V5.38; Groom *et al.*, 2016 ▸) for the 1,3,3-trimethyl-2-oxabi­cyclo­[2.2.2]octane (cineole) skeleton gave the parent structure (I) (ref code MOFPAY; Bond & Davies, 2001 ▸) and the oxidation products, 5,6-di­hydroxy­cineole (three steroisomers: ref codes DIFJAF, DIFJEJ and DIFJIN; Farlow *et al.*, 2013 ▸), 6-(1,3-dioxolan-2-yl)-5-ketocineole and 5-(1,3-dioxolan-2-yl)-6-ketocineole (ref codes DIFHOR and DIFHUX; Farlow *et al.*, 2013 ▸).

## Synthesis and crystallization   

1,8-Cineole (I) was mono-hy­droxy­lated using a recombinant *Escherichia coli* whole-cell fed-batch process using CYP101J2 in combination with suitable redox partner proteins from *S. yanoikuyae* B2 to provide a major product (Unterweger, 2016 ▸). The isolated material was further purified by recrystallization from diethyl ether/petroleum ether to afford white needles. The melting point (this work m.p. 371.2–371.8 K, lit. m.p. 371–372 K (Carman *et al.*, 1986 ▸), 370, 370, 369, 368, 371–372, 371–372, 371–372 369–372, 372 and 370 K as cited in Azerad (2014 ▸)) and ^1^H NMR spectrum are in agreement with cited literature values (Azerad, 2014 ▸) for either compound (IV) and/or (V). Optical rotation {this work [a]_D_ +32.0 (*c* 1.3, EtOH), lit [a]_D_ +31.9 (*c* 1.3, EtOH)}. The experimental data for the current material produced from the biotransformation of cineole is well aligned with one set of literature data (Carman *et al.*, 1986 ▸).

## Refinement   

Crystal data, data collection and structure refinement details are summarized in Table 2 ▸. H atoms potentially involved in hydrogen-bonding inter­actions were located by difference methods and were freely refined. Other H atoms were included in the refinement at calculated positions with C—H = 0.95–0.98 Å and treated as riding with *U*_iso_(H) = 1.2*U*_eq_(C) or 1.52*U*_eq_(O or methyl C).

## Supplementary Material

Crystal structure: contains datablock(s) I, global. DOI: 10.1107/S2056989017010015/hg5490sup1.cif

Structure factors: contains datablock(s) I. DOI: 10.1107/S2056989017010015/hg5490Isup2.hkl

Supporting information file. DOI: 10.1107/S2056989017010015/hg5490Isup3.cml

CCDC reference: 1560548

Additional supporting information:  crystallographic information; 3D view; checkCIF report

## Figures and Tables

**Table 1:** Hydrogen-bond geometry (Å, °)

*D*—H⋯*A*	*D*—H	H⋯*A*	*D*⋯*A*	*D*—H⋯*A*
O1—H1⋯O2^i^	0.80 (3)	1.97 (3)	2.7530 (19)	170 (3)

Symmetry code: (i) 
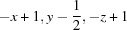
.

**Table 2:** Experimental details

Crystal data	
Chemical formula	C_10_H_18_O_2_
*M*_r_	170.24
Crystal system, space group	Monoclinic, *P*2_1_
Temperature (K)	123
*a*, *b*, *c* (Å)	6.3121 (1), 10.5611 (2), 7.9925 (2)
β (°)	112.126 (3)
*V* (Å^3^)	493.57 (2)
*Z*	2
Radiation type	Cu *K*α
μ (mm^−1^)	0.62
Crystal size (mm)	0.25 × 0.10 × 0.02

Data collection	
Diffractometer	Oxford Gemini Ultra CCD
Absorption correction	Multi-scan (*CrysAlis PRO*; Rigaku OD, 2015 ▸)
*T*_min_, *T*_max_	0.650, 1.000
No. of measured, independent and observed [*I* > 2σ(*I*)] reflections	6839, 1746, 1728
*R*_int_	0.027
(sin θ/λ)_max_ (Å^−1^)	0.596

Refinement	
*R*[*F* ^2^ > 2σ(*F* ^2^)], *wR*(*F* ^2^), *S*	0.029, 0.076, 1.05
No. of reflections	1746
No. of parameters	113
No. of restraints	1
H-atom treatment	H atoms treated by a mixture of independent and constrained refinement
Δρ_max_, Δρ_min_ (e Å^−3^)	0.13, −0.11
Absolute structure	Flack *x* determined using 804 quotients [(*I* ^+^)−(*I* ^−^)]/[(*I* ^+^)+(*I* ^−^)] (Parsons *et al.*, 2013 ▸)
Absolute structure parameter	0.07 (9)

Computer programs: *CrysAlis PRO* (Rigaku OD, 2015 ▸), *SHELXS97* (Sheldrick, 2015 ▸), *SHELXL2016* (Sheldrick, 2015 ▸), *X-SEED* (Barbour, 2001 ▸) and *publCIF* (Westrip, 2010 ▸).

## supplementary crystallographic information

### Crystal data

**Table:**

C_10_H_18_O_2_	*F*(000) = 188
*M**_r_* = 170.24	*D*_x_ = 1.146 Mg m^−^^3^
Monoclinic, *P*2_1_	Cu *K*α radiation, λ = 1.54184 Å
*a* = 6.3121 (1) Å	Cell parameters from 4695 reflections
*b* = 10.5611 (2) Å	θ = 7.6–66.8°
*c* = 7.9925 (2) Å	µ = 0.62 mm^−^^1^
β = 112.126 (3)°	*T* = 123 K
*V* = 493.57 (2) Å^3^	Plate, colourless
*Z* = 2	0.25 × 0.10 × 0.02 mm

### Data collection

**Table:**

Oxford Gemini Ultra CCD diffractometer	1746 independent reflections
Radiation source: fine focus sealed tube	1728 reflections with *I* > 2σ(*I*)
Mirror monochromator	*R*_int_ = 0.027
Detector resolution: 10.3389 pixels mm^-1^	θ_max_ = 66.7°, θ_min_ = 7.6°
ω scans	*h* = −7→7
Absorption correction: multi-scan (CrysAlis PRO; Rigaku OD, 2015)	*k* = −12→12
*T*_min_ = 0.650, *T*_max_ = 1.000	*l* = −9→9
6839 measured reflections	

### Refinement

**Table:**

Refinement on *F*^2^	Hydrogen site location: mixed
Least-squares matrix: full	H atoms treated by a mixture of independent and constrained refinement
*R*[*F*^2^ > 2σ(*F*^2^)] = 0.029	*w* = 1/[σ^2^(*F*_o_^2^) + (0.0408*P*)^2^ + 0.077*P*] where *P* = (*F*_o_^2^ + 2*F*_c_^2^)/3
*wR*(*F*^2^) = 0.076	(Δ/σ)_max_ < 0.001
*S* = 1.05	Δρ_max_ = 0.13 e Å^−^^3^
1746 reflections	Δρ_min_ = −0.11 e Å^−^^3^
113 parameters	Absolute structure: Flack *x* determined using 804 quotients [(*I*^+^)-(*I*^-^)]/[(*I*^+^)+(*I*^-^)] (Parsons *et al.*, 2013)
1 restraint	Absolute structure parameter: 0.07 (9)

### Special details

**Table:**

Geometry. All esds (except the esd in the dihedral angle between two l.s. planes) are estimated using the full covariance matrix. The cell esds are taken into account individually in the estimation of esds in distances, angles and torsion angles; correlations between esds in cell parameters are only used when they are defined by crystal symmetry. An approximate (isotropic) treatment of cell esds is used for estimating esds involving l.s. planes.

### Fractional atomic coordinates and isotropic or equivalent isotropic displacement parameters (Å^2^)

**Table:**

	*x*	*y*	*z*	*U*_iso_*/*U*_eq_	
O1	0.3440 (2)	0.06028 (14)	0.5824 (2)	0.0344 (4)	
H1	0.421 (5)	0.000 (3)	0.584 (4)	0.049 (8)*	
C1	0.3290 (3)	0.28323 (16)	0.5241 (2)	0.0245 (4)	
O2	0.3425 (2)	0.37248 (12)	0.39090 (15)	0.0273 (3)	
C2	0.3671 (3)	0.15220 (17)	0.4598 (2)	0.0268 (4)	
H2	0.525368	0.147597	0.459797	0.032*	
C3	0.1930 (3)	0.13171 (17)	0.2663 (3)	0.0327 (5)	
H3A	0.273802	0.126032	0.181704	0.039*	
H3B	0.109504	0.051339	0.259698	0.039*	
C4	0.0233 (3)	0.24262 (19)	0.2122 (3)	0.0311 (4)	
H4	−0.095250	0.227897	0.088837	0.037*	
C5	−0.0898 (3)	0.2491 (2)	0.3515 (3)	0.0357 (5)	
H5A	−0.152629	0.165173	0.363326	0.043*	
H5B	−0.217014	0.310951	0.312108	0.043*	
C6	0.0917 (3)	0.28989 (18)	0.5342 (3)	0.0299 (4)	
H6A	0.060505	0.377427	0.562909	0.036*	
H6B	0.085833	0.233283	0.631090	0.036*	
C7	0.1531 (3)	0.36548 (19)	0.2146 (2)	0.0277 (4)	
C8	0.0064 (4)	0.4834 (2)	0.1933 (3)	0.0394 (5)	
H8A	−0.121668	0.480975	0.076148	0.059*	
H8B	−0.052847	0.486220	0.290291	0.059*	
H8C	0.099284	0.558972	0.199665	0.059*	
C9	0.2605 (3)	0.3681 (2)	0.0727 (2)	0.0360 (4)	
H9A	0.139808	0.363569	−0.048097	0.054*	
H9B	0.347117	0.446788	0.084261	0.054*	
H9C	0.363568	0.295535	0.090656	0.054*	
C10	0.5167 (4)	0.3200 (2)	0.7013 (3)	0.0377 (5)	
H10A	0.515033	0.262157	0.796667	0.056*	
H10B	0.665254	0.314862	0.689156	0.056*	
H10C	0.491166	0.406855	0.732737	0.056*	

### Atomic displacement parameters (Å^2^)

**Table:**

	*U*^11^	*U*^22^	*U*^33^	*U*^12^	*U*^13^	*U*^23^
O1	0.0373 (8)	0.0291 (7)	0.0455 (8)	0.0094 (6)	0.0253 (7)	0.0102 (6)
C1	0.0236 (9)	0.0271 (10)	0.0230 (8)	−0.0020 (7)	0.0089 (7)	0.0028 (7)
O2	0.0264 (6)	0.0293 (7)	0.0244 (6)	−0.0054 (5)	0.0076 (5)	0.0019 (6)
C2	0.0226 (8)	0.0290 (9)	0.0315 (9)	0.0034 (7)	0.0133 (7)	0.0045 (8)
C3	0.0341 (10)	0.0280 (11)	0.0349 (10)	−0.0016 (8)	0.0116 (8)	−0.0065 (8)
C4	0.0226 (8)	0.0352 (11)	0.0303 (9)	−0.0013 (7)	0.0041 (7)	−0.0033 (8)
C5	0.0217 (9)	0.0414 (11)	0.0444 (11)	0.0002 (8)	0.0128 (8)	0.0045 (9)
C6	0.0300 (9)	0.0296 (10)	0.0357 (9)	0.0051 (7)	0.0187 (8)	0.0028 (8)
C7	0.0253 (8)	0.0329 (9)	0.0223 (8)	0.0028 (8)	0.0061 (6)	−0.0001 (8)
C8	0.0438 (12)	0.0399 (12)	0.0381 (11)	0.0136 (9)	0.0194 (10)	0.0101 (9)
C9	0.0347 (9)	0.0462 (11)	0.0279 (9)	0.0039 (10)	0.0127 (7)	0.0026 (9)
C10	0.0380 (11)	0.0445 (12)	0.0266 (9)	−0.0091 (9)	0.0078 (8)	0.0018 (8)

### Geometric parameters (Å, º)

**Table:**

O1—C2	1.425 (2)	C5—H5A	0.9900
O1—H1	0.80 (3)	C5—H5B	0.9900
C1—O2	1.448 (2)	C6—H6A	0.9900
C1—C10	1.515 (2)	C6—H6B	0.9900
C1—C2	1.526 (2)	C7—C8	1.523 (3)
C1—C6	1.532 (2)	C7—C9	1.525 (3)
O2—C7	1.4665 (18)	C8—H8A	0.9800
C2—C3	1.539 (3)	C8—H8B	0.9800
C2—H2	1.0000	C8—H8C	0.9800
C3—C4	1.535 (3)	C9—H9A	0.9800
C3—H3A	0.9900	C9—H9B	0.9800
C3—H3B	0.9900	C9—H9C	0.9800
C4—C7	1.531 (3)	C10—H10A	0.9800
C4—C5	1.534 (3)	C10—H10B	0.9800
C4—H4	1.0000	C10—H10C	0.9800
C5—C6	1.540 (3)		
			
C2—O1—H1	109 (2)	H5A—C5—H5B	108.4
O2—C1—C10	106.18 (14)	C1—C6—C5	109.29 (14)
O2—C1—C2	106.40 (13)	C1—C6—H6A	109.8
C10—C1—C2	112.35 (16)	C5—C6—H6A	109.8
O2—C1—C6	109.76 (13)	C1—C6—H6B	109.8
C10—C1—C6	112.00 (15)	C5—C6—H6B	109.8
C2—C1—C6	109.91 (14)	H6A—C6—H6B	108.3
C1—O2—C7	114.93 (12)	O2—C7—C8	107.90 (15)
O1—C2—C1	108.43 (14)	O2—C7—C9	106.51 (13)
O1—C2—C3	112.08 (15)	C8—C7—C9	108.91 (16)
C1—C2—C3	108.81 (14)	O2—C7—C4	107.07 (14)
O1—C2—H2	109.2	C8—C7—C4	113.06 (15)
C1—C2—H2	109.2	C9—C7—C4	113.05 (16)
C3—C2—H2	109.2	C7—C8—H8A	109.5
C4—C3—C2	109.53 (15)	C7—C8—H8B	109.5
C4—C3—H3A	109.8	H8A—C8—H8B	109.5
C2—C3—H3A	109.8	C7—C8—H8C	109.5
C4—C3—H3B	109.8	H8A—C8—H8C	109.5
C2—C3—H3B	109.8	H8B—C8—H8C	109.5
H3A—C3—H3B	108.2	C7—C9—H9A	109.5
C7—C4—C5	110.26 (16)	C7—C9—H9B	109.5
C7—C4—C3	109.25 (15)	H9A—C9—H9B	109.5
C5—C4—C3	107.20 (16)	C7—C9—H9C	109.5
C7—C4—H4	110.0	H9A—C9—H9C	109.5
C5—C4—H4	110.0	H9B—C9—H9C	109.5
C3—C4—H4	110.0	C1—C10—H10A	109.5
C4—C5—C6	108.51 (14)	C1—C10—H10B	109.5
C4—C5—H5A	110.0	H10A—C10—H10B	109.5
C6—C5—H5A	110.0	C1—C10—H10C	109.5
C4—C5—H5B	110.0	H10A—C10—H10C	109.5
C6—C5—H5B	110.0	H10B—C10—H10C	109.5
			
C10—C1—O2—C7	−171.24 (15)	C3—C4—C5—C6	68.1 (2)
C2—C1—O2—C7	68.89 (16)	O2—C1—C6—C5	63.37 (18)
C6—C1—O2—C7	−50.00 (18)	C10—C1—C6—C5	−178.95 (17)
O2—C1—C2—O1	−177.12 (13)	C2—C1—C6—C5	−53.32 (19)
C10—C1—C2—O1	67.09 (18)	C4—C5—C6—C1	−11.6 (2)
C6—C1—C2—O1	−58.34 (18)	C1—O2—C7—C8	109.18 (16)
O2—C1—C2—C3	−54.97 (17)	C1—O2—C7—C9	−134.02 (16)
C10—C1—C2—C3	−170.76 (15)	C1—O2—C7—C4	−12.81 (18)
C6—C1—C2—C3	63.81 (17)	C5—C4—C7—O2	65.78 (18)
O1—C2—C3—C4	113.35 (17)	C3—C4—C7—O2	−51.77 (18)
C1—C2—C3—C4	−6.56 (19)	C5—C4—C7—C8	−52.9 (2)
C2—C3—C4—C7	61.95 (19)	C3—C4—C7—C8	−170.47 (16)
C2—C3—C4—C5	−57.52 (19)	C5—C4—C7—C9	−177.24 (15)
C7—C4—C5—C6	−50.8 (2)	C3—C4—C7—C9	65.2 (2)

### Hydrogen-bond geometry (Å, º)

**Table:**

*D*—H···*A*	*D*—H	H···*A*	*D*···*A*	*D*—H···*A*
O1—H1···O2^i^	0.80 (3)	1.97 (3)	2.7530 (19)	170 (3)

Symmetry code: (i) −*x*+1, *y*−1/2, −*z*+1.
